# Acetone Applications in the Relief of Ulcerating Cancer

**Published:** 1909-07-03

**Authors:** 


### ACETONE APPLICATIONS IN THE RELIEF OF ULCERATING CANCER.

Acetone is a very hygroscopic liquid, so that it has been used in histological work for the hardening and preparation of tissues for microscopical purposes. The fact that acetone causes tissues to shrink and harden has suggested to Gellhorn, Maier, and others that it might be used in the relief of ulcerating cancers. This idea has been confirmed by actual experience. It is not claimed that the acetone constitutes in any way a cure for cancer, but it affords a comparatively simple way of relieving the fœtor, hæmorrhage, and discharge from an ulcerating growth. Dealing with inoperable uterine cancers, for instance, Maier finds that, whereas patients who are left alone suffer from loss of strength owing to the recurrent hæmorrhage, the foulness of the vaginal discharge, and the fœtor of the atmosphere in which they are compelled to live, the use of acetone almost immediately stops both bleeding and discharge, and thereby leads to cessation of the fœtor also. Health improves in consequence of this, appetite returns, and the patient, though incurable perhaps, is greatly relieved. The only symptom that may not be benefited is the local pain, if there be any; this is generally neither increased nor diminished.

The procedure in the case of inoperable cancer of the cervix is so simple that it can be carried out in the patient's own home or in the consulting-room. With or without a preliminary curettage, a Ferguson's speculum is introduced into the vagina, the position of the patient being such that the speculum points upwards so that fluid poured into it does not at once flow out again. The full Trendelenburg posture may not be necessary, but the patient should be upon her back with her shoulders down and her pelvis raised. The application causes no pain, therefore no anæsthetic is needed. The speculum is held down around the cervix, so that no fluid poured into it can escape into the vagina outside the speculum. Even should any do so there is no great risk, for no pain results, and all that happens is that the surface of the mucosa where the acetone has touched it becomes temporarily whitened. From four to eight drachms of acetone are poured into the speculum and left there in contact with the growth for from a quarter to a half an hour. The fluid is then swabbed out, the speculum is removed, the interior of the vagina is also swabbed as a precautionary measure, and nothing more is done for a week. Though the first application will have checked the fœtor, hæmorrhage, and discharge, the application naturally needs to be repeated; and as a rule the best plan is to perform precisely the same action once a week, twice in ten days, or even twice a week regularly. The relief amply repays the trouble taken.

